# Evaluation of cinnamon extract effects on *clbB* gene expression and biofilm formation in *Escherichia coli* strains isolated from colon cancer patients

**DOI:** 10.1186/s12885-020-06736-1

**Published:** 2020-03-30

**Authors:** Faezah Kosari, Mohammad Taheri, Abbas Moradi, Reza Hakimi Alni, Mohammad Yousef Alikhani

**Affiliations:** 1 Microbiology Department, Faculty of Medicine, Hamadan University of Medical Sciences, P.O box: 6517838678, Hamadan, Iran; 2 Department of Community Medicine, Hamadan University of Medical Sciences, Hamadan, Iran; 3 Department of Pathobiology, Faculty of Veterinary Science, Bu-Ali Sina University, Hamadan, Iran; 4 Brucellosis Research Center, Hamadan University of Medical Sciences, Hamadan, Iran

**Keywords:** *E. coli*, Cinnamaldehyde, Biofilm, *Pks*, *clbB*, Colon cancer

## Abstract

**Background:**

Colon cancer is one of the most common malignancies and the fourth leading cause of cancer-related mortality in the world. Colibactin, which is synthesized by the *pks* genomic island of *E. coli* interfere with the eukaryotic cell cycle. Cinnamon has an antimicrobial effect and considered as a colon cancer-preventing agent. The aim of the study was to evaluate the effects of cinnamon extract and cinnamaldehyde on *clbB* gene expression and biofilm formation in clinical isolates of *E. coli*.

**Methods:**

Thirty *E. coli* carrying *pks* gene were isolated from the colon cancer patients, inflammatory bowel disease and healthy subjects. Antibiotic susceptibility was evaluated by disk diffusion method and the minimum inhibitory concentration of cinnamon essential oil and cinnamaldehyde by microdilution broth method. In vitro biofilm formation of *E.coli* isolates was monitored using a microtiter plate method. The presence of *clbB, clbA* and *clbQ* genes in *E.coli* isolates were evaluated by PCR. The effect of cinnamaldehyde and cinnamon essential oil on *clbB* gene expression was evaluated by Real-Time PCR.

**Results:**

The highest antibiotic resistance was obtained with 94.4% for ticarcillin-clavulanic acid, azithromycin, amoxicillin, and amikacin. The MIC for all clinical isolates was 32 μl/ml of cinnamon essential oil and the MIC of cinnamaldehyde was between 0.00002 to 0.03 μl/ml. After exposure of isolates to cinnamon extract and cinnamaldehyde, 40 and 13.3% were weakly biofilm producers, respectively. The frequencies of *clbB, clbA,* and *clbQ* genes were 23.3, 23.3, and 26.7%, respectively. The expression of *clbB* gene in the presence of the Sub-MIC concentration of cinnamon essential oil and cinnamaldehyde was decreased in 8 isolates compared to untreated isolates (*p*-value < 0.05).

**Conclusions:**

The antibacterial activity of cinnamaldehyde and cinnamon essential oil allows the use of these herbal compounds for treatment or supplements in infections caused by *E. coli* and in patients with suspected colorectal cancer.

## Background

Colorectal cancer (CRC) is one of the most common cancers and causes of death in the world. Because of its high incidence and mortality rate, colorectal cancer is a major public health problem. There is increasing evidence that the mucosa-associated flora and their related products may be important in the pathogenesis of inflammatory bowel diseases, ulcerative colitis, and colorectal cancer via various mechanisms [1, 2]. In gastrointestinal diseases, the rate of attachment of *E. coli* to intestinal mucus is increased in the ileum and colon [3, 4]. However, these patients have high levels of *E. coli* belong to B2 phylogroup, which induces more expression of *CEACAM6*, a cancer marker, in the intestinal epithelial cells and intensifies the inflammation [5].

Some *E. coli* strains carry a combination of virulence genes that disrupt the intestinal microbial balance and can cause intra and extra-intestinal infections [6]. Pathogenic *E. coli* strains synthesize various virulence factors, including several toxins called cyclomodulins such as cytolethal distending toxins (CDT), cytotoxic necrotizing factor (CNF), cycle inhibiting factor, and colibactin. Recent studies have shown that cyclomodulin producing strains are among the B2 phylogenetic group which is more prevalent in people with colorectal cancer [7]. Colibactin is a peptide encoded by the *pks* genomic islands, causing DNA double-strand breaks and chromosomal instability in human cells. The efficacy of colibactin and its expression requires bacterial contact with the host cell [8].

In the last decade, increased drug resistance is considered as the most important barrier to successful treatment of infectious diseases and the control of the pathogenicity of microbial agents [9]. The development of new and natural antimicrobial agents due to increased drug resistance in bacterial pathogens is increasing. The cinnamon essential oil contains important compounds including cinnamaldehyde, eugenol, caryophyllene, linalool, alpha-terpineol, coumarin, cineol, and terpinene [10]. Cinnamon has antifungal and antibacterial properties that are related to the cinnamaldehyde. Cinnamaldehyde is an aromatic aldehyde compound and a major component of cinnamon extract (about 65%). The main advantage of cinnamaldehyde is that it does not need direct contact as antimicrobial activity and classified as a GRAS molecule by the US Food and Drug Administration and approved for use in food [11]. The antimicrobial effects of cinnamon have been proven in various studies [12]. The aim of this study was to investigate the effect of cinnamon and its essential oil (Cinnamaldehyde) on *pks* gene expression and microbial biofilm formation of *E.coli* strains isolated from colon cancer patients, inflammatory bowel disease and healthy subjects.

## Methods

### Sample collection

This study was a cross-sectional study and the *E. coli* strains isolated from the patients which CRC, inflammatory bowel disease and healthy subjects, during a period from July 2016 to August 2018 in Hamadan, west of Iran. Thirty *pks* positive *E. coli* were collected from colon biopsy specimens of colorectal cancer (13 specimens), inflammatory bowel disease (8 specimens) and healthy subjects (9 specimens).

### Culture condition and isolation of *E. coli*

Biopsy specimens were taken from 50 to 100 mg and were immediately placed in a tube containing 100 ml of sterile phosphate buffer saline (PBS) and transferred to the microbiology laboratory. The biopsy specimens were then washed three times in the laboratory with 10 ml of PBS and centrifuge at 900 g for 5 min. After homogenous and washing, the samples were cultured on blood agar and MacConkey agar using a sterile loop and incubated at 37 °C for 24 h. Bacterial strains were confirmed using conventional methods and stored at − 20 °C [13].

### Antibiotic susceptibility test

Antibiotic susceptibility to ciprofloxacin (5 μg), imipenem (10 μg), meropenem (10 μg), ticarcillin-clavulanic acid (75/10 μg), co-amoxiclav (20/10 μg), amoxicillin (10 μg), amikacin (30 μg), piperacillin (100 *μ*g), ceftazidime (30 *μ*g), trimethoprim-sulfamethoxazole (1.25/23.75 μg), and gentamicin (10 μg) was performed bythe Kirby-Bauer disk diffusion method according to Clinical and Laboratory Standard Institute guidelines (CLSI). *Escherichia coli* ATCC 25922 was used as quality control strains [14].

### Biofilm production assay

Biofilm production abilities of isolated strains were quantified by the microtiter plate method as previously described using 1% crystal violet [15]. The absorbance was measured at 620 nm. Each assay was performed in triplicate and the results were reported as mean ± SD. Tryptic Soy Broth (TSB) medium without bacteria was used as a negative control.

### Minimal inhibition concentration

Minimum inhibitory concentrations (MICs) of cinnamon extract and cinnamaldehyde (Kimia Gostar Research Company, Tehran, Iran) were determined by the broth microdilution method in 96-well plates. Serial concentrations of cinnamon extract and cinnamaldehyde were used. MIC was considered as the last well in which no turbidity was observed. *Escherichia coli* ATCC 25922 was used as quality control strains.

### Detection of pks genes

The genomic DNA of bacteria was extracted from overnight cultures of *E.coli* isolates using the boiling method. All isolates were screened for the presence of the *pks* encoding genes, including *clbB, clbA* and *clbQ* using a single PCR technique. The list of primers [16, 17] used in the present study has been shown in Table 1.

**Table 1:** The primer sequences used in this study

Genes	Primer sequences (5′---3′)	Fragment size (bp)	Reference
*clbB-F* *clbB-R*	GCGCATCCTCAAGAGTAAATA GCGCTCTATGCTCATCAACC	283	[16]
*clbQ –F* *clbQ -R*	GCAC GATCGGACAGGTTAAT TAGTCTCGGAGGGATCATGG	308	[16]
*clbA-F* *clbA-R*	AAGCCGTATCCTGCTCAAAA GCTTCTTTGAGCGTCCACAT	342	[16]
*pks*	TCGATATAGTCACGCCACCA GTCAAGCGAGCATACGAACA	733	[17]
*16srRNA*	GGTGAATACGTTCCCGG TACGGCTACCTTGTTACGACTT	144	[16]

The PCR reaction mixture contained 1 μL (10 pmol) of each primer, 2 μL DNA, 25 μL PCR Master Mix in a final 50 μL reaction volume. DNA amplification was conducted in a thermal cycler (Bio-Rad, USA), under the following conditions: initial denaturation at 94 °C for 5 min, followed by 35 cycles of denaturation at 94 °C for 30 s, an annealing temperature for each gene according to Table 1 for 40 s, an extension at 72 °C for 50 s, followed by a final extension at 72 °C for 5 min. Electrophoresis of the amplified DNA fragments, along with a 100 bp DNA ladder, was carried out using 2% agarose gel in TBE buffer.

### RNA isolation and quantitative real-time RT-PCR

In order to investigate the gene expression of *clbB* gene, quantitative Real-Time PCR was performed at sub-MIC concentrations of cinnamon extract and cinnamaldehyde. Total RNA was extracted using the Trisol solution. The concentration and optical absorbance of each extracted RNA were confirmed with Nanodrop (Epoch Microplate Spectrophotometer) at 260 nm/280 nm. RNA samples were stored at − 80 °C. In order to elicit the DNA contamination from total isolated RNA, each sample was treated with DNaseI kit (Fermentase Co., USA). The cDNA was synthesized according to the cDNA synthesis kit (Takara, Japan) according to the manufacturer’s instruction. RT-PCR was performed in order to cDNA synthesis confirmation. Q-PCR was performed by the SYBR Green gene expression assay (AMPLIQON Co, Denmark) with the Roch system. The primers used in this study were listed in Table 1 and the *16 s rRNA* gene was used as the housekeeping gene. In addition to the melting curve analysis, the specificity of each primer was confirmed by DNA sequencing of Real-Time PCR product. The sample contains all of the PCR mixtures except template used as negative template control (NTC). Thermal program was performed by the following steps; 1) the first denaturation was optimized at 95 °C for 10 min, 2) secondary denaturation was set at 95 °C for 15 s, 3) annealing temperature was set to 60 °C for 1 min and finally, 4) 72 °C was optimized as incubation time for 1 min, step 2–4 was repeated for 40 cycles. Each experiment was repeated three times.

### Data analysis

Statistical analysis was performed using SPSS 24 software. Chi-square and Fischer tests were used to compare group variables. The MIC of cinnamon and cinnamaldehyde was analyzed using Mann-Whitney. A real-time PCR graph was drawn using Graph pad prism software.

## Result

In this study, a total of 30 clinical isolates of *pks* positive *E. coli* were studied. Thirteen, and eight strains isolated from colorectal cancer and inflammatory bowel disease individuals, respectively, and other isolates related to healthy subjects.

### Frequency of antibiotic resistance in *pks* positive *E.coli* isolates

**Fig. 1:**
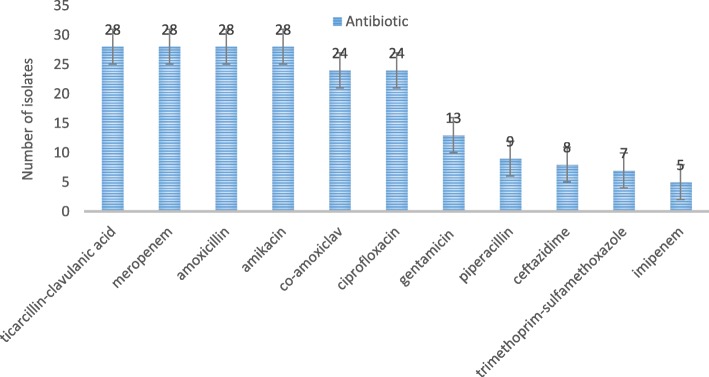
Frequency of resistant *E. coli* isolates to different antibiotics

The minimum inhibitory concentration (MIC) for 30 clinical isolates of *E. coli* was 32 μl per ml of cinnamon, with no significant difference between isolates. For cinnamaldehyde active ingredient of cinnamon, the maximum value of MIC was 0.015 μl/ml (30.8%), and 0.0005 μl/ml (37.5%) and 0.015 μl/ml (30%) was observed in colorectal cancer, inflammatory and healthy isolates, respectively. The MIC for *E. coli* ATCC 25922 was 0.0000001 μL/ml. According to the results of Fig. 1, the highest antibiotic resistance was related to the antibiotics ticarcillin/clavulanic acid, meropenem, amoxicillin and amikacin (93.4%) and the least to imipenem (16.7%) and trimethoprim-sulfamethoxazole (23.4%). The results showed that there was no significant difference between susceptibility and antibiotic resistance of the isolates according to the type of the sample (*p*>0.05).

### Frequency of *clbB*, *clbQ* and *clbA* genes in *pks* positive *E. coli* isolates

**Fig. 2:**
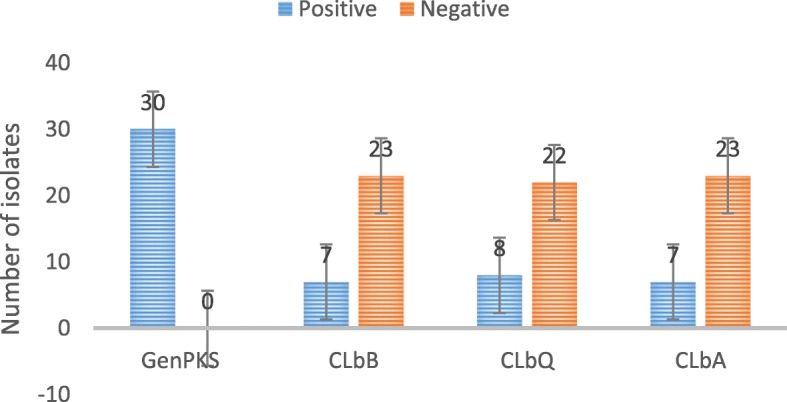
Frequency of clbB, clbQ and clbA genes in the pks positive *E. coli* isolates Positive: The presence of the specific genes in the isolates; Negative: The absence of the specific genes in the isolates.

All thirty *E. coli* isolates were *pks* positive and the frequency of *clbB*, *clbQ* and *clbA* were 23.4, 26.7, and 23.4%, respectively. The frequency of *clbB*, *clbQ* and *clbA* positive genes in *pks* positive isolates was not statistically significant (Fig. 2). The results showed that there was no significant difference between the antibiotic susceptibility of the isolates (p>0.05). There was no statistically significant difference between the frequency of *clbB*, *clbQ* and *clbA* positive genes in the *pks* positive *E. coli* isolates by sample type (Fig. 2).

### Frequency of microbial biofilm before and after treatment with cinnamon extract and cinnamaldehyde

**Fig. 3:**
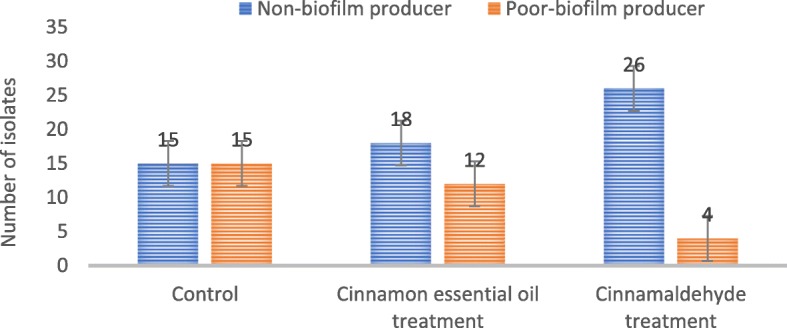
Frequency of Biofilm producing *E. coli* isolates after exposure to cinnamaldehyde and cinnamon essential oil

The results showed, 50% of the isolates were weakly biofilm producers and 50% were unable to produce biofilms. After exposure of isolates to cinnamon extract and cinnamaldehyde, 40 and 13.3% were weakly biofilm producers, respectively (Fig. 3).

### Effect of cinnamaldehyde and cinnamon extract on *clbB* gene expression in *E.coli* isolates

**Fig. 4:**
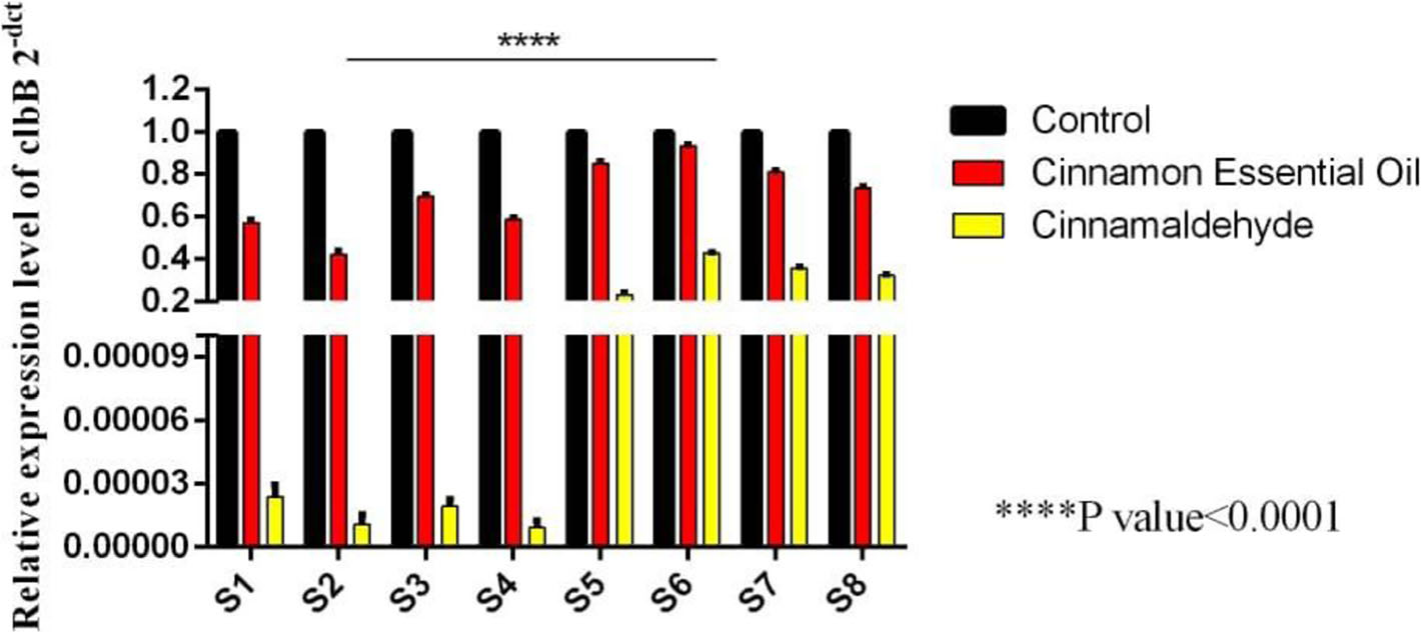
Comparative expression of clbB gene after treatment with cinnamon and cinnamaldehyde essential oils in samples S1-S8

The effect of the Sub-MIC concentration of cinnamaldehyde on *clbB* gene expression was evaluated for eight isolates. The *16sRNA* gene expression was measured as an internal control. *clbB* gene expression was significantly higher in Sub-MIC cinnamaldehyde compared to untreated samples in eight isolates. The four isolates (S1-S4) showed drastically reduced *clbB* gene expression (Table 2, Fig. 4).

In the *E. coli* isolates treated with cinnamon essential oil, the expression of *clbB* gene was slightly decreased compared to the control sample, which was also significant (Table 3).

**Table 2:** The effect of cinnamaldehyde on *clbB* gene expression in *E.coli* isolates

isolates	***E***.***coli isolates*** source	Sub MIC (μl/ml)	Mean of CT 16sRNA	Mean of CT ***clbB***	Mean of CT ***clbB Treat***	Fold change		Result
1	Cancer	0.0002	14. 23	18.45	34.05	***clbB***	0.000018656	Down
						**Control**	1. 000	
2	Cancer	0.001	14. 14	19.34	36.02	***clbB***	0.0000058222	Down
						**Control**	1. 000	
3	Cancer	0.015	14. 33	20.99	34.02	***clbB***	0. 000117098	Down
						**Control**	1. 000	
4	Inflammation	0.015	14. 59	20.52	38.01	***clbB***	0.000005585	Down
						**Control**	1. 000	
5	Inflammation	0.03	14. 31	32.14	34.3	***clbB***	0. 236,514,412	Down
						**Control**	1. 000	
6	Healthy	0.03	14. 25	31.12	32.21	***clbB***	0. 432,268,616	Down
						**Control**	1. 000	
7	Healthy	0.03	14. 36	33.1	34.73	***clbB***	0. 283,220,971	Down
						**Control**	1. 000	
8	Inflammation	0.06	14. 87	34.76	36.98	***clbB***	0. 230,046,913	Down
						**Control**	1. 000

**Table 3:** The effect of cinnamon essence on *clbB* gene expression in *E.coli* isolates

isolates	***E.coli*** source	SubMIC (μl/ml)	Mean of CT 16sRNA	Mean of CT ***clbB***	Mean of CT ***clbB Treat***	Fold change		Result
1	Cancer	0.0002	14. 54	18.45	19. 5	***clbB***	0. 554,784,736	DOWN
						**Control**	1. 000	
2	Cancer	0.001	14. 61	19.34	20. 41	***clbB***	0. 40,332,088	DOWN
						**Control**	1. 000	
3	Cancer	0.015	14. 32	20.99	21. 5	***clbB***	0. 683,020,128	DOWN
						**Control**	1. 000	
4	Inflammation	0.015	14. 23	20.52	21	***clbB***	0. 574,349,177	DOWN
						**Control**	1. 000	
5	Inflammation	0.03	14. 72	32.14	32. 88	***clbB***	0. 840,896,415	DOWN
						**Control**	1. 000	
6	Healthy	0.03	14. 37	31.12	31. 01	***clbB***	0. 779,228,237	DOWN
						**Control**	1. 000	
7	Healthy	0.03	14. 55	33.1	33. 42	***clbB***	0. 801,069,878	DOWN
						**Control**	1. 000	
8	Inflammation	0.06	14. 41	34.76	34. 86	***clbB***	0. 726,986,259	DOWN
						**Control**	1. 000	

## Discussion

Bacterial infections have long been established as important factors in the etiology of several human cancers. Increased drug resistance is considered as the most important barrier to successful treatment of infectious diseases and the control of the pathogenicity of microbial agents. This investigation presents evidence for the effect of cinnamon extract and its active ingredient cinnamaldehyde on *clbB* gene expression and microbial biofilm formation of *E. coli* strains isolated from colon cancer patients.

Based on the finding of the present study, the number of our cancer samples was 47.3%, and the prevalence of *pks* gene in the *E. coli* isolates was 100%. Our results are in agreement with previous investigations. A study conducted by Suresh et al., in 2018 on the genetic and molecular function of the *pks* gene in 462 *E. coli* intestinal pathogens showed that 35 (7.6%) isolates had *pks* genes, 97% of which were pathogenic and biofilm production was strong in 21 *pks* -positive isolates [18]. It was also found that 11% of these isolates have multidrug resistance which is involved in colibactin resistance. A review study conducted in 2019 by Sadeghi et al. investigated the antimicrobial activity of cinnamaldehyde. They found that cinnamaldehyde, alone or in combination with other plant extracts, had a good antioxidant, antimicrobial and anticancer function. Its anticancer property is due to its effect on the via gene in cancer cells, which has been shown to be in our study the anticancer property of cinnamaldehyde [19]. A study in 2019 by Gilling et al. On the antimicrobial effect of essential oils and extracts on *E. coli*, examined 11 extracts and essential oils including cinnamaldehyde, which showed a significant antimicrobial effect on *E. coli*. In this study, the effects of the essential oils were greater than the extracts, and they also increased the susceptibility of the bacteria to antibiotics. According to the results of two studies showed that cinnamaldehyde had a significant antimicrobial effect on *E. coli* and increased antibiotic susceptibility of this bacterium [20].

Another study was conducted in 2018 by Mohamed et al. on the antibacterial and antibiotic effect of cinnamaldehyde on *Acinetobacter baumannii*, biofilm production was 86.95% strong, 52.17% moderate and 39.17% poor. They observed that cinnamaldehyde at low concentrations also had antimicrobial activity against *A. baumannii* and the best antibiofilm activity at MIC was 1.2 and 1.4 ppm, which reduced biofilm production by 18.5%. They found that cinnamaldehyde had antimicrobial activity against this bacterium, which in our study showed the antimicrobial effects of cinnamaldehyde; However, in our study, the rate of biofilm reduction was higher than that obtained in this study, and the MIC was lower than ours in this study [21]. In 2015 study by Kim et al. in Korea, showed that 0.01% cinnamon essential oil inhibited 96% of biofilm formation in *Pseudomonas aeruginosa*. Also, at this concentration of cinnamon extract, the expression of adhesion and Shiga-toxin production genes in *E. coli* was reduced up to three times. But in our study, cinnamon extract (60%) and cinnamaldehyde (86.7%) inhibited biofilm production. In the last study, gene expression was also decreased, but the type of genes studied in our research was different from that of Kim et al. [22]. In India, Aparna et al. reported that a concentration of 8–10 mg/ml of the cinnamon aqueous extract showed a 44% antibacterial effect against *E. coli*, inhibiting 6.8% toxin production and inhibiting protease production [23]. In 2012, Packiavathy et al. investigated the effect of methanolic extract of cinnamon on the inhibition of biofilm formation and QS system inhibition in *P. aeruginosa*. The results of this study showed that 4 mg/ml methanol extract of cinnamon was the lowest concentration that inhibited bacterial growth. Lower concentrations of MIC inhibited the movement and subsequently inhibited the formation of biofilm by the bacterium so that at a concentration of 2 mg/ml of cinnamon methanol extract inhibited the early stages of biofilm formation in the bacteria under QS system control [24].

In 2019, Malekpour et al. conducted a mixed effect of cinnamon and clove essential oils on *E. coli* isolates containing some broad-spectrum beta-lactamase enzymes, which was different from our study [25]. They did not show any growth in 20 strains at concentrations of 1600 ppm and 3200 ppm of cinnamon and clove essential oils, however, in two isolates, MIC of cinnamon essential oil was 400 and 800 ppm and in one isolate, MIC of clove essential oil of 1600 ppm. They also showed that the two essential oils had a synergetic effect on the *E. coli* isolates containing the broad-spectrum beta-lactamase TEM gene. In our study, the lowest MIC was 0.00003 ppm for cinnamaldehyde and 31.25 ppm for extract. The results obtained from this study are in good harmony with the most similar studies [21, 23].

## Conclusion

In conclusion, cinnamon extract and cinnamaldehyde have a good antibacterial effect on *E. coli* and can reduce biofilm production and expression of genes that are effective in causing disease. *E. coli* is one of the most active bacteria causing colorectal cancer that *pks* gene plays a significant role in this disease. Real-time PCR can be a gold-standard method for diagnosis. Cinnamon essential oil and active ingredient have various medicinal and therapeutic properties that can reduce the antibiotic resistance of germs, including *E. coli.*

## Data Availability

The datasets used and/or analyzed during the current study available from the corresponding author on reasonable request.

## Abbreviations

CRC: Colorectal cancer
*E. coli*: *Escherichia coli*
PBS: Phosphate buffer saline
CLSI: Clinical and Laboratory Standard Institute guidelines
TSB: Tryptic Soy Broth
MICs: Minimum inhibitory concentrations
PCR: Polymerase chain reaction
Q-PCR: Real-time RT-PCR
